# Non-contact elastography methods in mechanobiology: a point of view

**DOI:** 10.1007/s00249-021-01567-9

**Published:** 2021-08-31

**Authors:** Silvia Caponi, Alessandra Passeri, Giulio Capponi, Daniele Fioretto, Massimo Vassalli, Maurizio Mattarelli

**Affiliations:** 1 Istituto Officina dei Materiali, National Research Council (IOM-CNR), Unit of Perugia, c/o Department of Physics and Geology, University of Perugia, 06123 Perugia, Italy; 2 Department of Physics and Geology, University of Perugia, 06123 Perugia, Italy; 3 CEMIN-Center of Excellence for Innovative Nanostructured Material, 06123 Perugia, Italy; 4 James Watt School of Engineering, University of Glasgow, Glasgow, UK

**Keywords:** Mechanobiology, Bioimaging, Elastography, Brillouin spectroscopy

## Abstract

In recent decades, mechanobiology has emerged as a novel perspective in the context of basic biomedical research. It is now widely recognized that living cells respond not only to chemical stimuli (for example drugs), but they are also able to decipher mechanical cues, such as the rigidity of the underlying matrix or the presence of shear forces. Probing the viscoelastic properties of cells and their local microenvironment with sub-micrometer resolution is required to study this complex interplay and dig deeper into the mechanobiology of single cells. Current approaches to measure mechanical properties of adherent cells mainly rely on the exploitation of miniaturized indenters, to poke single cells while measuring the corresponding deformation. This method provides a neat implementation of the everyday approach to measure mechanical properties of a material, but it typically results in a very low throughput and invasive experimental protocol, poorly translatable towards three-dimensional living tissues and biological constructs. To overcome the main limitations of nanoindentation experiments, a radical paradigm change is foreseen, adopting next generation contact-less methods to measure mechanical properties of biological samples with sub-cell resolution. Here we briefly introduce the field of single cell mechanical characterization, and we concentrate on a promising high resolution optical elastography technique, Brillouin spectroscopy. This non-contact technique is rapidly emerging as a potential breakthrough innovation in biomechanics, but the application to single cells is still in its infancy.

In everyday life, to understand how hard or soft a material is, we touch it and observe the induced deformation. This is the basic concept for physical measurements of elastic properties, which are expressed in terms of elastic moduli, linking a stress (force divided by unit area) to its corresponding strain (deformation normalized by the size of the object). Elastic moduli are significant parameters in material science but, their key importance has been widely recognized also in understanding structures and functionality of biological systems: mechanics affects biology at a fundamental level from single molecules to tissue growth and development (Wang et al. 2009), and living cells have evolved an exquisite ability to sense both elastic and viscous components of the microenvironment (Cantini et al. 2019).

The gold standard for measuring nanoscale mechanics of biological materials and single cells is currently considered the Atomic Force Microscope (AFM) (Garcia 2020). This technique implements a micrometric version of standard indentation experiments, pushing on a substrate with an elastic cantilever and measuring the corresponding deformation of the sample. Standard AFM cantilevers are typically terminated with a sharp conical tip, with a curvature radius in the range from few nm to 50–100 nm. However, cantilevers with a spherical micrometric tip (colloidal probes) are often adopted to measure mechanical properties of single cells, to avoid puncturing them but also to offer a well-controlled interaction geometry (Dörig et al. 2013). To convert the force–distance curve obtained with the AFM in a meaningful mechanical parameter, the theoretical framework developed for contact mechanics has been specialized for the nanoscale (Garcia 2020). This methodology has offered a number of interesting insights into the role of cell mechanics in central biological problems, such as for cancer (Stylianou et al. 2018), liver disease (Baldini et al. 2019) or neurodegenerative disorders (Jazvinšćak Jembrek et al. 2015), to cite some. Nevertheless, the process to obtain viscoelastic properties from AFM measurements relies on the adoption of a-priori models, which requires a complex analysis which is extremely sensitive to the specific experimental conditions (Haase and Pelling 2015; Lüchtefeld et al. 2020). Other nanotechnology tools have been adopted to study the mechanical properties of single cells, including optical tweezers (Arbore et al. 2019), acoustic force spectroscopy (Sorkin et al. 2018), and magnetic tweezers (Kilinc and Lee 2014). A broad comparison of methods for cell mechanics has been recently carried out, showing that different methods might provide differences up to two orders of magnitude in the evaluation of the same elastic modulus (Wu et al. 2018a). This striking result clearly highlights that the measurement of cell mechanics based on deformation devices is highly dependent on the pattern of force application, both for inherent technical caveats and for the emergence of apparent stiffening phenomena in the material (Ciccone et al. 2020).

The relevance of the interplay of mechanical factors with structure and functionality in living cells is nowadays clear, and many key connections have been highlighted between mechanical alterations and the emergence of diseases at tissue level. There is a growing interest to translate this concept towards pre-clinical research, but this requires a technological boost, developing reliable non-invasive tools able to characterize the viscoelastic properties of living materials with unprecedented resolution.

On the other side, micro-elastography methods are being tested to specifically address clinical needs (Kennedy et al. 2020), pushing the resolution of traditional medical imaging techniques. In medical elastography, acoustic waves are injected in tissues by vibrating devices and the deformations induced by the mechanical load are evaluated using either acoustic or electromagnetic probes (Kennedy et al. 2017). The spatial resolution and penetration depth of these techniques depend on the features of the exciting field and detection method. In particular, the size of the smallest element which can be imaged is inversely related with the frequency of the field (Strohm et al. 2013), but so is the penetration (Qian et al. 2017). The typical resolution of the apparata used in clinical applications is of the order of 100 µm (frequency in the MHz range), which is associated to a penetration depth in the mm range. In order to increase the resolution, with the scope of studying not only tissues but also cells, higher frequencies are needed. Scanning acoustic microscopy employing piezoelectric transducers (frequency < 1 GHz) reaches a resolution in the micron range (Qian et al. 2017) while the use of laser-generated acoustic waves allows study of the system response to THz acoustic waves reaching a spatial resolution up to a few tens of nanometers (Dehoux et al. 2015). A major drawback of these last techniques is that the imaged cells need to be fixated on a suitable substrate, both for keeping them stable below the acoustic pressure field and for transmitting the acoustic pulse in the case of laser generation.

While innovations in medical elastography have demonstrated an enormous potential for this technique in the clinical setting (Selvaraj et al. 2021), they need to introduce external stresses or strains—and indirectly extract viscoelastic properties of the material—and currently available devices still do not reach the cellular resolution required to address mechanobiological questions. In a different approach to elastography, the inherent acoustic field of the sample can be probed, without the need to inject an external sound wave (Fig. 1). In fact, in any given material, spontaneously propagating acoustic waves are always present. These spontaneous vibrational modes, called acoustic phonons, present themselves as pressure (density) waves, (Longitudinal Acoustic waves) or shear waves (Transverse Acoustic waves). Both types of perturbation give rise to a modulation of the material refractive index that can be probed by means of an external electromagnetic field, where the features of the external field select the characteristics of the observed phonons (Berne and Pecora 1976).

**Fig. 1:**
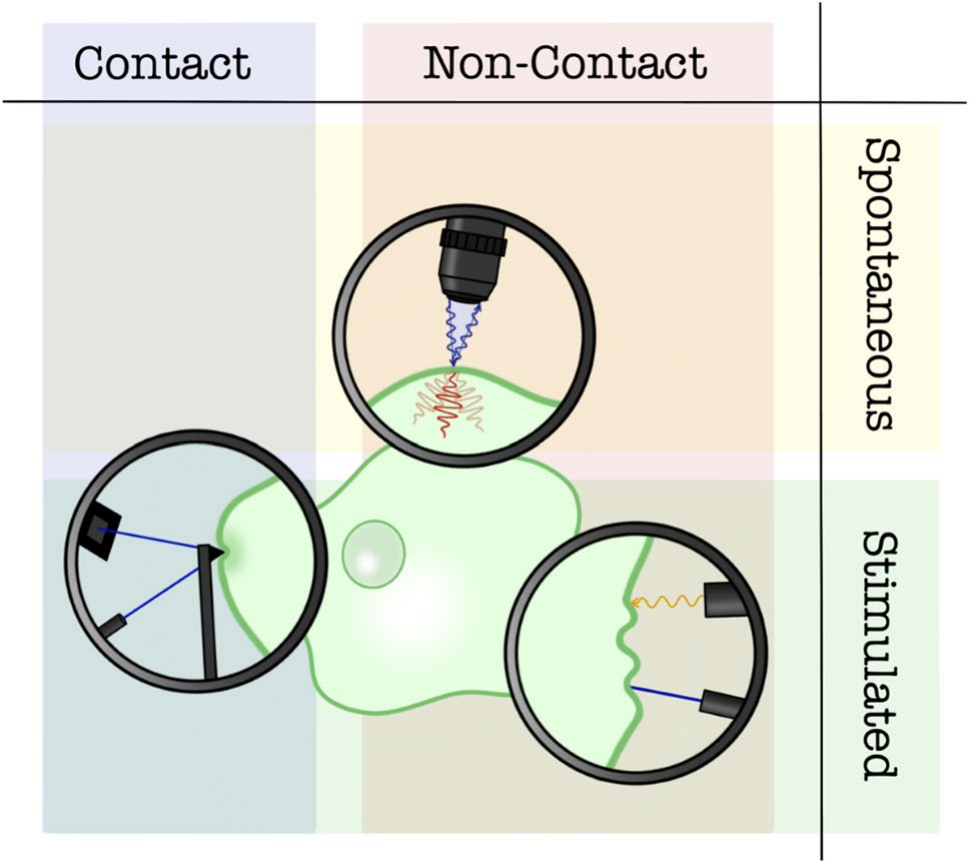
A general breakdown of the techniques used to probe the mechanical properties of cells and tissues, discriminated on the basis of the need to come into contact with the sample (such as AFM) and the need to produce a mechanical stimulus. Spontaneous BS is unique in that it is based on sensing thermally activated acoustic modes

Brillouin spectroscopy (BS) observes these spontaneous vibrations through the interaction with a (low power) laser beam. Its easy applicability, which does not require an actual contact for the mechanical testing, enabled the characterization of a broad class of materials including crystalline solids and amorphous materials, including under extreme conditions of pressure or temperature. The only operational requirement is the availability of a free path to illuminate the sample with a monochromatic beam and to analyze the frequency shift of the scattered light. In fact, due to the interaction with phonons, a tiny fraction (less than 10^−10^) of the incident light is scattered at different wavelengths, the difference depending on the sound velocity, *v*, in the material. As the speed of sound is directly related to the elastic modulus (*M* = *v*^2^*ρ*, *ρ* being the density) this technique characterizes in a non-contact, non-invasive and label-free condition the mechanical properties of materials. The frequency of the probed phonons determines the frequency at which the mechanical properties are investigated. In particular, using a visible laser beam the phonon frequency is in the 1–30 GHz range in polymers, hydrogels and in most biological materials (Mattana et al. 2018; Mercatelli et al. 2019; Margueritat et al. 2019; Cardinali et al. 2019; Palombo and Fioretto 2019; Bailey et al. 2020; Prevedel et al. 2019). It is noteworthy that as the biological materials have a viscoelastic nature, the absolute value of the measured mechanical properties can differ when probed at different frequencies (Mattana et al. 2017; Scarcelli et al. 2015a). This effect has been well-observed by nanoindentation or microrheology measurements, even for small changes of frequency in the Hz region (Wu et al. 2018b). Increasing the stress frequency, from Hz to kHz to MHz, the elastic moduli are usually larger as the system is less able to follow the applied stress through structural rearrangement (Giudice et al. 2017). Indeed, this phenomenon causes also the apparent change of elastic properties observed when applying a fixed stress for longer times (Efremov et al. 2019). BS probing the system in the GHz region is at the extreme high-frequency range of these techniques and the measured longitudinal elastic moduli are always in the GPa range.

Also, the achievable spatial resolution is strictly related to the propagation properties of the investigated phonon. At Brillouin frequencies it can reach the micrometric scale (Mattarelli et al. 2020; Caponi et al. 2020). This makes it important to reduce the observed volume to enable discrimination of the micromechanical properties of heterogeneous materials, as most biological materials are. Coupling the Brillouin interferometer with a confocal microscope, it is possible to achieve sub-cellular spatial resolution. Scanning the laser spot on the sample, high resolution 3D images with mechanical contrast can be collected. In recent publications, Brillouin imaging demonstrated its ability to probe the mechanical modulations of sub-cellular components (Mattana et al. 2018; Antonacci et al. 2018; Scarcelli et al. 2015b; Antonacci and Braakman 2016) and of tissue structures (Mercatelli et al. 2019; Elsayad et al. 2016; Bevilacqua et al. 2019; Schlüßler et al. 2018; Raghunathan et al. 2017). Its sensitivity to mechanical properties together with its contactless features makes Brillouin imaging a reliable tool for biological and biomedical applications (Shao et al. 2019). Figure 2 shows two remarkable applications of the technique, where Brillouin spectroscopy was used to evidence variations of the elastic properties in different kind of tissues, both hard such as femoral bones (Cardinali et al. 2020) and soft such as the cornea (Mercatelli et al. 2019), where since the sample is transparent, BS allows 3D maps to be obtained.

**Fig. 2:**
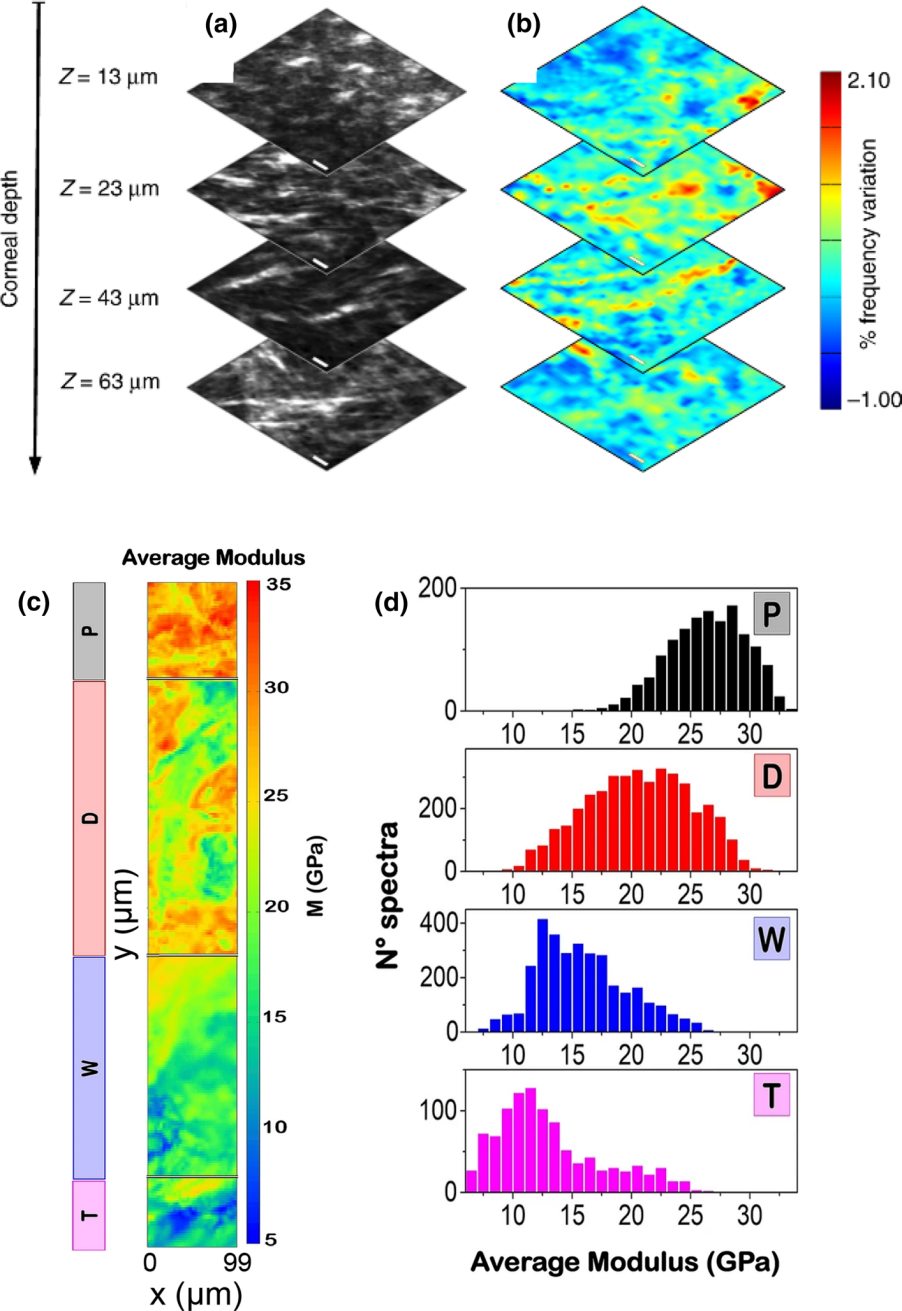
Top: Correlative Second Harmonic Generation (SHG)-Brillouin axial optical sectioning analysis on corneal stroma. Comparison between **a** SHG images, **b** relative variations of Brillouin frequency, acquired at the specified depths below Bowman’s membrane. The reference frequency is ~ 9 GHz. Scale bars 10 μm (adapted from Mercatelli et al. 2019). Bottom: Brillouin imaging of bones. **c** Average mechanical properties, collected in 2D maps, with 3 μm steps, over the 4 main regions of the diaphysal ring of a human femur. **d** Distribution of point averaged longitudinal elastic moduli (adapted from Cardinali et al. 2020)

The great potential of the BS technique attracted the attention of the mechanobiology community, opening a critical discussion of the possibility (and meaningfulness) of comparing Brillouin mechanical characterization with de facto standards, such as AFM, or other quasi-static methods already used. In some materials, a sort of correlation has emerged between the longitudinal elastic modulus *M*, measured by Brillouin spectroscopy and the Young modulus, *E*, measured in low frequency regime (Scarcelli et al. 2015a; Meng et al. 2017). However, it has to be considered that this relation is only empirical and strongly sample dependent. In fact, at any given frequency, two independent mechanical parameters (like *E* and *M*) are needed to describe the elastic properties at equilibrium, even considering only a pure elastic response of a homogeneous and isotropic material (Alam and Garra 2020). Therefore, from a fundamental point of view, it is not possible to establish a universal constitutive relation linking *M* and *E* without some a-priori assumption on one other elastic parameter such as shear modulus, *G*; Bulk modulus, *K*, or Poisson’s ratio, *ν*. Indeed, in different biological materials the ratio between *M* and *E* can span many orders of magnitude (it is in the order of a unit for hard tissues such as bones and about 10^6^ for soft tissues such as liver or brain) as they measure the uniaxial strain response of the system to a uniaxial stress with and without constraints in the transverse expansion, so that they essentially indicate how the material opposes a change in volume and density (*M*) or a change in shape (*E*) (Mattana et al. 2017; Guimarães et al. 2020). This means that solid-like and liquid-like samples will have similar *M*, as both are incompressible, but very different *E*, because liquids only can easily change shape (Emelianov et al. 2006). Moreover, it has to be considered that the two techniques probe the material at different frequency (~ kHz vs GHz). In the presence of relaxation processes with characteristic frequencies intermediate between those of the probes, which is often the case in biological materials, this causes the peculiar effect that the sample behaves as a liquid at low frequency and as a solid at the higher frequency probed by BS.

These fundamental reasons prevent the existence of a direct link between the measurement by AFM and BS. Nevertheless, both techniques open a window on the complex viscoelastic nature of the probed sample, and the local modulation of the measured mechanical quantities in space can be converted into a meaningful map of “mechanical contrast” which can convey a physiologically relevant message. This is why in many cases, evaluating the relative changes of the elastic modulus is an extremely effective strategy, eventually more informative and surely more robust than providing its absolute value (Baldini et al. 2019).

Accordingly, the micromechanical modifications probed by Brillouin have been used to evaluate the effectiveness of drugs in tumor spheroids (Margueritat et al. 2019), in phenotyping healthy and tumor cells (Mattana et al. 2018), in the evaluation of nuclear mechanics within intact cells (Zhang et al.  2017, 2020) and of nuclear softening during transendothelial migration (Roberts et al. 2021). These few examples, in which the capability offered by micro-Brillouin spectroscopy was used in the mechanobiology field, clarify the advantage of the technique for non-contact deep 3D mechanical characterization. Indeed, as no local contact is needed, either for detecting the signal nor for inducing the stress field, this technique is fully suited to operate in experimental conditions mimicking the in vivo situation, where physical access to the cells or to tissues cannot be assured. There are a few issues that need to be addressed to allow widespread use of this technique, which are the analysis of turbid media and the laser power/acquisition time, still not able to provide the high-throughput investigation of living cells. In the near future, we expect the recognized potentials to guide research to develop new technological solutions and improve the theoretical framework for an effective application in the biological as well as in the biomedical field.

## Data Availability

Not applicable.
